# Identifying enhanced cortico-basal ganglia loops associated with prolonged dance training

**DOI:** 10.1038/srep10271

**Published:** 2015-06-02

**Authors:** Gujing Li, Hui He, Mengting Huang, Xingxing Zhang, Jing Lu, Yongxiu Lai, Cheng Luo, Dezhong Yao

**Affiliations:** 1 Key Laboratory for NeuroInformation of Ministry of Education, School of Life Science and Technology, University of Electronic Science and Technology of China.

## Abstract

Studies have revealed that prolonged, specialized training combined with higher cognitive conditioning induces enhanced brain alternation. In particular, dancers with long-term dance experience exhibit superior motor control and integration with their sensorimotor networks. However, little is known about the functional connectivity patterns of spontaneous intrinsic activities in the sensorimotor network of dancers. Our study examined the functional connectivity density (FCD) of dancers with a mean period of over 10 years of dance training in contrast with a matched non-dancer group without formal dance training using resting-state fMRI scans. FCD was mapped and analyzed, and the functional connectivity (FC) analyses were then performed based on the difference of FCD. Compared to the non-dancers, the dancers exhibited significantly increased FCD in the precentral gyri, postcentral gyri and bilateral putamen. Furthermore, the results of the FC analysis revealed enhanced connections between the middle cingulate cortex and the bilateral putamen and between the precentral and the postcentral gyri. All findings indicated an enhanced functional integration in the cortico-basal ganglia loops that govern motor control and integration in dancers. These findings might reflect improved sensorimotor function for the dancers consequent to long-term dance training.

Dance is a complex rhythmic sensorimotor activity that integrates organized spatial pattern body movements, temporally synchronized with timekeepers and other dancers, and adheres to a trajectory map of the body in exocentric space^1^,^2^. In other words, dance is a sophisticated activity that humans are capable of executing through streamlining and negotiating a combination of perception, cognition and action within the human brain. There is considerable interest among neuroscientists to discover the neural basis for dance and to understand how it affects the human brain. This study concerned the positive effects of dance training and how it could benefit dancers in terms of overall neural function.

There are two core elements in dance: metrical movement and organized spatial patterns^1^,^2^. The former is defined as temporal synchronization, which means the match of one’s movements to the beat of music. The latter focuses on the body movements of the dancer, which are organized into accurate spatial position as dictated by the standards of this art. When dancers perform their routines, the brain goes through a course of processing and integration of sensorimotor and spatial information relative to the body followed by coordination with the musical rhythm. These properties are the ones that make the dancers’ cognitive conditioning different from similarly regimented individuals such as athletes^3^. In addition, dance is included in the interdisciplinary field of neuroaesthetics, which unites the various forms of artistic expression and the neuroscientific examination of how the human brain perceives, processes and executes various arts, such as dance^4^,^5^. Neuroaesthetics researchers have focused on how dance training affects the human mind in terms of the intrinsic workings of the human brain’s neural architecture and the forces underlying the coordinated patterns of activity that support the thought, reasoning, action, and emotion that are involved in dance^4^,^6^,^7^,^8^,^9^,^10^. As it specifically pertains to dance, neuroaesthetics serves as a new means by which neuroscience researchers can investigate the integration of the sensorimotor functions^2^, elements of aesthetics^9^,^11^ and emotion^12^ that arise from dance.

Using neuroimaging, brain activity during dance performance has been observed in the superior temporal gyri (reflects the auditory processing of music), the basal ganglia (reflecting the processing of metrical movements), the thalamus (relating to the connection between somatosensory and motor parameters), and the sensorimotor areas (M1, S1, premotor and SMA)^1^,^10^,^11^. Particularly, functional MRI (fMRI) studies have demonstrated the many effects of dance on the human brain^13^,^14^. For example, the sensorimotor characteristics of dance movement comprise a well-attended topic. Relevant studies have investigated the neuronal mechanisms of small-scale spatial patterns in dance, such as finger tapping^15^,^16^, limb coordination^17^,^18^ and gait control^19^. However, those studies have been focused on simple, individual movements that are very basic and are evident in nearly all types of human behaviors. In fact, the realization of dance requires that the brain employ not only the execution of motor components but also the integration of sensorimotor parameters, such as muscle dynamics, body posture, musical rhythm and movement speed. Therefore, researchers have studied how the brain organizes and integrates sensorimotor parameters when dance is proceeding^1^,^2^,^7^. These studies have revealed that the basal ganglia, somatotropic areas, premotor regions and SMA regions, which are in the sensorimotor network, are highly involved in the dance process. Particularly, from those findings, we can see an important involvement of cortico-basal ganglia loops in dance, which are likely to integrate various motor functions and induce smoothly executed, goal-directed behaviors^20^.The findings from these studies have indicated which specific parts of the brain are responsible for processing sensorimotor activity and the energy levels required during such actions.

Resting-state functional connectivity (rs-FC), which is based on the association of intrinsic spontaneous low-frequency blood-oxygenation-level dependent (BOLD) signal fluctuations between spatially remote brain regions, is an excellent approach with which to study brain plasticity in humans^21^,^22^. Prior studies have used rs-FC to investigate training-related functional differences in the brains of people who are highly trained in certain skills, such as musicians^23^,^24^,^25^ and athletes^26^. Recently with the development of functional connectivity methods, functional connectivity density (FCD) has become a new data-driven method by which to effectively characterize voxel-wise short- and long-range functional connectivity in the human brain^27^. This method overcomes the limitation of seed-based approaches for identifying and locating functional hubs in the brain.

Based on prior studies, this study aimed to show that dancers had better motor control and better-integrated sensorimotor system when compared with non-dancers. We thus hypothesized that the dancers might show increased functional connections in sensorimotor brain networks that are related to dance properties. As such, we designed a cross-sectional experiment (dancers and non-dancers) and used rs-FC to test our hypothesis. FCD analysis and rs-FC analysis were performed to assess functional differences in sensorimotor systems induced by dance training. The purpose of these analyses was to find evidence of enhancement and integration of the various parts of the human brain linked to the sensorimotor movement of the dancers, which might be associated with the long-term effects of engaging in dancing.

## Method and Materials

### Subjects

Two groups of test subjects were recruited for this study, including (1) 28 dancers (10 males and 18 females, *M*_age_ = 19.07 ± 1.82 yrs)-henceforth referred to as the “dancer group”-and 33 non-dancing individuals (12 males and 21 females, *M*_age_ = 19.48 ± 1.43 yrs)-the “non-dancer group.” The participants in the dancer group consisted of college students majoring in modern dance at the Southwest University for Nationalities and the University of Electronic Science and Technology of China (UESTC). All of the subjects were assessed by a group of senior dancers. Subjects who qualified according to the admission requirements for a professional modern dance student were defined as dancers. The dancers engaged in regular dance training for an average of 12 hours per week with training occurring year-round over an extended period of time ranging from 7-17 years. We excluded subjects whose training periods were too irregular as determined by a questionnaire survey. Details about the subjects in the dancer group are provided in Table 1. The non-dancer group consisted entirely of UESTC students lacking formal dance training; these participants were matched with the dancer group with respect to age, gender and level of education (for gender, *p *= 0.96, according to a chi-square test; for age, *p* = 0.33, according to two-sample *t*-tests; for education, *p* = 0.16, according to two-sample *t*-tests). All subjects were right-handed according to the Edinburgh Inventory^28^ and exhibited normal hearing, with no reported history of neurological illness. This study was approved by the UESTC Ethics Board, and every subject provided written consent to participate in this study. All the methods were carried out in accordance with the approved guidelines.

### Neuroimaging data acquisition

Images were acquired on a 3T MRI scanner (GE DISCOVERY MR750) at the MRI Research Center of UESTC. During scanning, we used foam padding and ear plugs to reduce head motion and scanning noise, respectively. Resting-state functional MRI data were acquired using gradient-echo echoplanar imaging(EPI) equences (repetition time [TR] = 2000 msec, echo time [TE] = 30 msec, flip angle [FA] = 90°, matrix = 64 × 64, field of view [FOV] = 24 × 24 cm^2^, slice thickness/gap = 4 mm/0.4 mm), with an eight-channel phased array head coil. All subjects underwent a 510-second resting-state scan to yield 255 volumes (32 slices per volume). None of the subjects performed a dance piece prior to the fMRI imaging. During resting-state fMRI, all subjects were instructed to be eye-closed and to move as little as possible without falling asleep. Subsequently, high-resolution T1-weighted images were acquired using a 3-dimensional fast spoiled gradient-echo (T1-3D FSPGR) sequence (TR = 6.008 msec, TE = 1.984 msec, FA = 9°, matrix = 256 × 256, FOV = 25.6 × 25.6 cm^2^, slice thickness (no gap) = 1 mm, 152 slices).

### fMRI preprocessing

Data processing was carried out using SPM8 (Statistical Parametric Mapping, http://www.fil.ion.ucl.ac.uk/spm/software/spm8) software. A series of preprocessing steps were performed: (1) discarding five volumes to establish the magnetization equilibrium; (2) slice-timing correction; (3) head-motion correction; (4) normalization of images with an EPI template in the Montreal Neurological Institute (MNI) atlas space 29 and resampling to 3 × 3 × 3 mm^3^; (5) temporal filtering (band-pass 0.01-0.08 Hz); and (6) regressing nuisance signals (six motion parameters). The general processing procedures of spatial smoothing (Gaussian kernel of a full-width at half-maximum (FWHM) of 8 mm) and regressing nuisance signals (white matter, cerebro-spinal fluid, global signal) were not included for the FCD analysis but were included for the functional connectivity analysis. Any subject who had a maximum displacement in any of the cardinal directions larger than 1.5 mm or a maximum spin larger than 1 degree were excluded from subsequent analyses. In addition, we also assessed translation and rotation in both groups using the following formula: 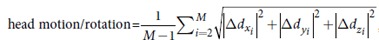, where M is the length of the time courses (*M* = 250 in this study). *x_i_*, *y_i_* and *z_i_* are translations/rotations at the *i*th time point in the x, y, and z directions, respectively, Δ*d_xi_* = *x_i_*–*x_i–1_*, and similar for *y_i_* and *z_i_*.

### Short- and long-range FCD

According to the approach introduced by Tomasi and Volkaw^27^,^30^, we used custom-written software to evaluate the individual voxel-vised FCD maps. The threshold (Tc) of the correlation coefficient to define the functional connection is a key parameter in FCD analysis. Based on prior knowledge^27^,^30^, the Tc value was set as 0.6. First, we obtained the short-range FCD using a “growing” algorithm. In this algorithm, for a given voxel *x*_0_, an additional voxel *x*_j_ was added to the list of neighbor voxels of *x*_0_, if *x*_j_ was adjacent to a voxel that was linked to *x*_0_ by a continuous path of functionally connected voxels and the correlation coefficient between *x*_0_ and *x*_j_ was larger than Tc. This calculation was repeated for all voxels that were adjacent to the neighbor voxels of clusters centered at *x*_0_ in an iterative manner until no new neighbors could be added to the list. The short-range FCD of *x*_0_ was defined as the number of elements in the list of neighbors. Then, we also calculated the long-range FCD, which was equated to the difference between global FCD and short-range FCD. The global FCD of a given voxel was defined as the number of functional connections (Tc > 0.6) between this voxel and all other voxels in the brain. Therefore, the short-range FCD of a given voxel reflected local functional connectivity, and the long-range FCD reflected functional connectivity with distant regions. Finally, individual normalized short-and long-range FCD (*k*-score) maps were obtained through division by the mean value of each individual map and spatial smoothing using 8 mm FWHM, respectively. Voxel-wised two-sample *t*-tests calculated in SPM8 were used to evaluate group differences in short- and long-range FCD maps between the groups.

### Functional connectivity analyses

We examined functional connectivity by taking the brain regions that had revealed different FCDs (pursuant to the FCD statistical tests between the two groups) as the seeds. The mean BOLD time series were extracted from the seeds. Subsequently, functional connectivity analysis was performed between the seed and all voxels in the brain. The resulting correlation coefficients were transformed to approximate a Gaussian distribution using Fisher’s *z*-transformation. In this manner, functional connectivity maps of these seeds were produced for each participant. Voxel-wise two-sample *t*-tests calculated in SPM8 were used to determine differences in functional connectivity of the seeds between the two groups. Additionally, based on the analysis of the FCD and FC, the region-wise functional connectivity analysis and classification method were used in the following analyses (Supplement).

### Correlations between functional properties and dance training variables

Pearson’s correlation analysis was used to investigate the relevance to dance learning of the altered functional brain properties of the dancer group. First, we correlated the dance-training variables (average training time per week, age upon the commencement of dance training, years of dance training) with the k-scores of the FCDs in regions of interest (ROI). For findings of functional connectivity of the seeds with different FCD regions, we extracted the average *z*-score of the ROI in which altered functional connectivity (FC) with those seeds was observed. Each ROI’s average z-score was correlated with the dance-training variables.

## Results

### Functional connectivity density analysis

None of the subjects was excluded according to the head-motion criterion. There were no significant differences between the two groups with regard to head motion or rotation (two-sample two-tailed t-tests: *T* = 1.31*, P* = 0.19 for translational motion, and *T* = 1.22, *P* = 0.23 for rotational motion).

**Figure 1:**
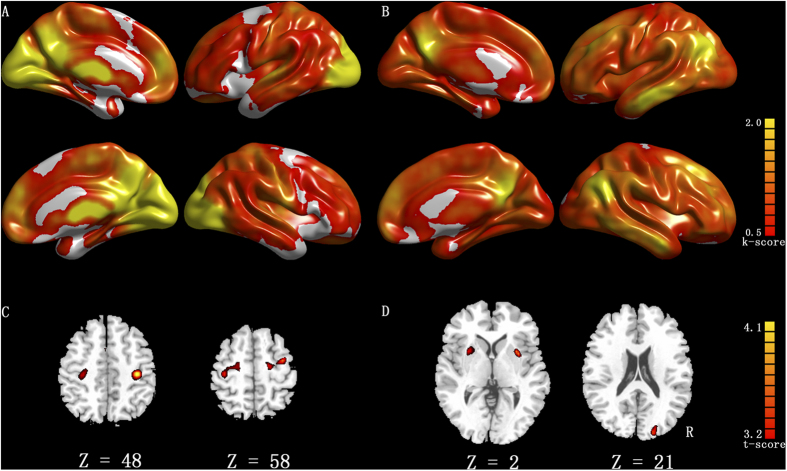
Result of the FCD analyses. Row A and row B show the spatial distribution of the average short-range FCDs and long-range FCDs, respectively, superimposed on the cerebral cortex for all subjects. Row C represents increased short-range FCDs of dancer group compared with non-dancer group (*p* < 0.001, cluster threshold *k* > 600 mm^3^). Row D represents increased long-range FCD of dancer group compared with non-dancer group (*p* < 0.001, cluster threshold *k *> 600 mm^3^).

The short- and long-range FCD maps were calculated for each individual. We detected similar patterns as observed in previous studies^27^,^30^ through the averaged FCD maps (Fig. 1A,B). The short- and long-range FCD patterns were observed at high magnitude in the bilateral posterior cingulate, occipital and prefrontal cortices. Compared with the non-dancers, the dancers principally exhibited increased short-range FCDs in the postcentral and the precentral gyri and increased long-range FCDs in the bilateral putamen and the occipital cortex (Table 2, Fig. 1C,D).

### Functional connectivity analyses of regions with different FCDs

**Figure 2:**
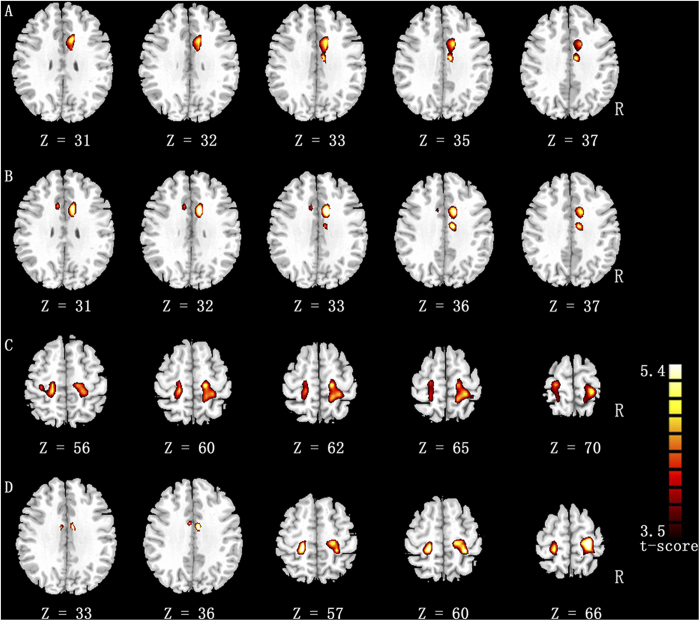
The enhanced functional connections in the dance group compared with the non-dancer group (p < 0.05, FDR-corrected, cluster threshold *k* > 600 mm^3^). Row ‘A’ represents the significantly increased functional connectivity with left putamen seed and row ‘**B**’ reveals that of the right putamen seed. Row ‘**C**’ represents the significantly increased functional connectivity with left precentral seed. Row ‘**D**’ reveals the significantly increased functional connectivity with right postcentral seed.

Short-range FCDs in the sensorimotor cortex (the bilateral precentral and postcentral gyri) and long-range FCDs in the bilateral putamen and right occipital cortex significantly increased. Through the use of seed-based functional connectivity analyses for five regions (the bilateral putamen, right occipital gyrus, right precentral gyrus, and left postcentral gyrus), we further investigated specific functional connectivity. Compared with the non-dancers, the dancers showed significantly increased functional connections between the putamen and the middle cingulate cortex (MCC), as well as increased sensorimotor region functional connectivity with the precentral gyri and the postcentral gyri (Table 3, Fig. 2). No different functional connection was found with the right occipital seed. Enhanced functional integration in the cortico-basal ganglia loops was found through the analysis of region-wise functional connectivity in the sensorimotor regions (Supplement). Furthermore, we found three biomarkers for optimal group differentiation in dancers and non-dancers by means of classification analysis (Supplement).

### Relationships between functional properties and dance-training variables

**Figure 3:**
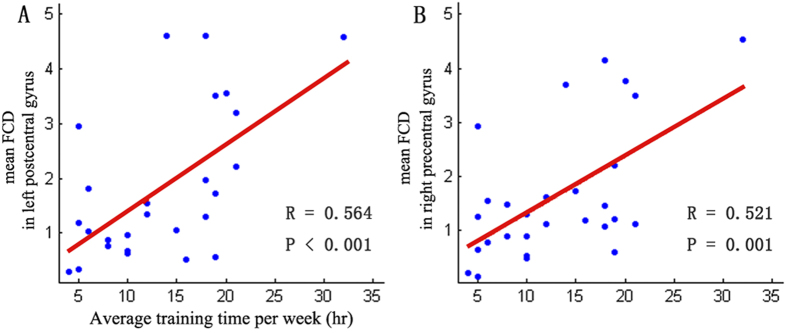
Positive relationships between functional properties and the average training time per week (hr). Relationships between the average training time per week and short-range FCD k- scores in left postcentral gyrus(**A**) and in right precentral gyrus(**B**).

In the dancer group, we found significant positive correlations between the average training time per week and mean FCD k-scores of ROIs (the left postcentral gyrus (Fig. 3A) and the right precentral gyrus (Fig. 3B)) with different short-range FCDs.

**Figure 4:**
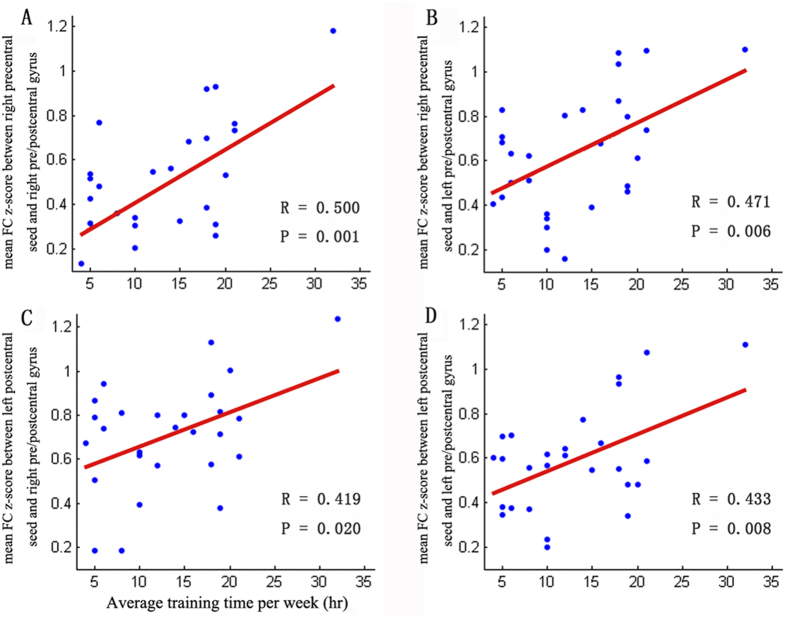
The relationship between the average training time per week and the mean FC *z*-score of the seed of the precentral areas: **A**” (right postcentral gyrus and precentral gyrus) and “**B**” (left postcentral gyrus and precentral gyrus). The relationship between average training time per week and the mean FC *z*-score of seed of left postcentral and its increased FC brain regions: “**C**” (right postcentral gyrus and precentral gyrus) and “**D**” (left postcentral gyrus and precentral gyrus). The abbreviation ‘hr’ represents hours.

There were 7 clusters that manifested significant connection differences with four seeds, as shown in Table 3. We found that the mean functional connectivity z-score between the right precentral seed and its increased functional-connectivity brain regions (the right precentral and postcentral gyri (Fig. 4A) and the left precentral and postcentral gyri (Fig. 4B)) were positively correlated with the average training time per week. We observed a significant positive correlation between the average training time per week and the mean *z*-score around the left postcentral seed and its increased functional-connectivity brain regions (the right precentral and postcentral gyri (Fig. 4C) and left precentral and postcentral gyri (Fig. 4D)). Functional connections positively correlating with average training time existed in sensorimotor regions. There was no significant correlation between functional properties and dance-training variables (i.e., the age at which dance training commenced and the years of dance training).

## Discussion

Our findings identified improved functional connectivity in cortico-basal loops in the brains of dancers who had gone through intensive dance training for years. First, we found significantly increased short-range FCDs in the primary sensorimotor cortex and long-range FCDs in the bilateral putamen in dancers compared with non-dancers. Second, the dancers demonstrated significantly increased putamen functional connectivity with the MCC and connectivity in the sensorimotor cortices. Finally, the behavioral data analysis revealed positive relationships between functional properties and the average training time per week. These findings provided evidence supporting the notion that long-term dance training may be related to enhanced functional integration in cortico-basal ganglia loops.

### Improved motor execution and learning

Similar to the regimented motor training required of athletes, dance training includes massive motor execution. When dance movements are performed, internal processing of kinesthesia occurs, and increased use of the muscles is actuated by the sensorimotor cortex. We found increased short-range FCDs and enhanced functional connectivity in the sensorimotor cortices (i.e., the bilateral postcentral gyri and the bilateral precentral gyuri in dancers. The sensorimotor cortices, including primary motor cortex (M1) and primary somatosensory cortex (S1), which take charge of the forces responsible for muscle contraction in initial and final positions in dance and in basic sensorimotor activities, serve as areas important in motor execution^1^. In fact, the M1 and S1 are relatively efficient in motor implementation because they are reciprocally connected both anatomically and functionally^31^. Furthermore, the M1 and S1 are highly participative in complex sequential sensorimotor movements, such as complex finger tapping^15^, gait control^19^ and limb coordination^17^,^32^. In addition to motor execution, the ability to learn new movements is clearly pronounced in dancers whose livelihood depends on rapid and adept movement production and reproduction^6^. Specifically, a dancer learns a sequence of movements by tracing gait patterns, repeating movements, and following others dancers who are demonstrating movements. In the process, two types of motor learning are applied: the acquisition of new spatiotemporal muscle-activation patterns and motor adaptation^33^. The former can occur when a dancer learns a novel set of body motions for a piece of music. The latter can be considered a perfect match between sensory input and motor performance with or without the circumstance of music in dance. Intriguingly, the sensorimotor area manifests a predominantly involvement in motor learning, including movement repetition and practice^34^,^35^, movement-sequence learning^36^,^37^ and sensorimotor-association learning^38^,^39^,^33^. Hence, consistent with these perspectives on motor execution and learning, our results supported the enhanced integration of sensorimotor regions in dancers, although the findings would be similar to those conducted for other motor trainings. Moreover, we found significantly positive correlations between average training time per week and functional properties. Dancers with 10 years of training can be identified as allocating increased attention to sensory experiences because of their internal processing of kinesthesia-i.e., knowing where their bodies are in space. Specifically, these dancers were trained in modern dance; therefore, they often danced with partners. Thus, there would be even more opportunities by which to enhance sensorimotor connectivity through such experiences. Therefore, we speculate that this improved local functional connectivity of sensorimotor cortices reflects improved motor execution and learning consequent to dancing expertise.

### Improved motor control and integration

Different from regimented motor training, dance training can lead to profound changes in the functional organization of the sensorimotor cortices because of the need for artistic fine motor skills^40^. Additionally, elaborate motor control and integration involves more brain regions except for S1 and M1. Some studies have shown functional and structural improvement in the sensorimotor network among people who learn and acquire particular motor skills—e.g., pianists^36^, violinists^41^, and dancers^2^. In our study, we found that enhanced short- and long-range FCDs existed in the cortico-basal ganglia loops for dancer group, including in the postcentral gyri, the precentral gyri, the bilateral putamen and the occipital cortex. Furthermore, we found significantly increased functional connectivities among these regions (Fig. 2). All of these regions with enhanced functional connectivities in dancers were involved in cortico-basal ganglia loops. These loops are constantly concerned with the performance of various motor function^42^ and especially govern motor control and integration^20^, which refer to the anatomical and functional links between the basal ganglia and diverse motor-related cortex areas, such as the cingulate motor areas, the primary motor cortex, and the supplementary and presupplementary motor areas^43^,^44^. In particular, we found that the MCC manifested enhanced connections with the bilateral putamen and M1 (see Supplementary), supported by the close anatomical and functional connections between the cingulate motor area and the putamen^43^,^45^,^46^. Previous neuroscientific studies of dance have reported a series of changes in these loops. For example, Brown *et al.* reported enhanced activations in this loop in dancers and presumed that these activations might help with the organization and integration of segments of action and interactions among entrainment, meter and spatial patterning in dance^1^. In addition to the functional findings, structural neuroplasticities have been found in the cortico-basal ganglia loops of dancers, including decreased gray matter volume in left premotor cortex, SMA, putamen, and superior frontal gyri^2^. Meanwhile, specific illness involving disturbed motor function, such as Parkinson’s disease, exhibits decline in the loops^47^. The increasing functional connectivity we found in the loops might relate to the process and integration of motor parameters and sensory information because dance involves the control of simple movements and integrates opponent movements to whole-body rhythmic movements, which demands intensive neuron motor control and integration. Former relevant studies have reported that there is, accordingly, plasticity within and between disparate cortical regions and the subcortical nuclei^2^,^4^. These parts work together to process motor information and realize the control and integration of action^1^,^7^. Therefore, our findings might reflect greater sensorimotor control and integration in dancers.

### The putamen and the rhythm of dance

Most dance is performed to musical rhythms. The entrainment of dance to musical rhythms involves not only coherence from a temporal perspective but also a synchronization to the motor pattern in metric music bits, which is a unique trait of dance^1^. In our study, we found an enhanced long-rang FCD and FC in the bilateral dorsal anterior putamen in dancers. In general, the dorsal anterior putamen revealed patterns of connectivity with frontal regions implicated in executive function control^48^. The metric elementary movement patterns were strongly and closely related to the putamen^49^,^50^. For example, rhythmic finger tapping was associated with involvement of the putamen^15^,^49^,^50^,^51^, and a metric foot flexion/extension activated the putamen^19^. Similar findings with dance studies have indicated that the putamen is involved in the voluntary control of metric movements in dance. In previous studies, the structural and functional changes induced by metric movements have been observed in the bilateral putamen in dancers^1^,^2^. In our study, we found an enhanced long-rang FCD and FC in the bilateral dorsal anterior putamen, in accord with Brown’s result. Because our results mirror those of earlier studies, it might be explained that dance training made dancers repeat timed and regular movement patterns (to a metric music bit) over an extended period of time; therefore, dancers might have enhanced connectivity in the putamen compared with those who have not engaged in such experiences.

### Limitations

Some limitation of the study should be mentioned. On one hand, the threshold (Tc) of the correlation coefficient to define the functional connection was set at 0.6 in the FCD analysis based on prior knowledge, and this fixed value may lead to some false positive or negative findings. Flexible thresholds, such as a set of continuous thresholds, should be considered in the future. On the other hand, the differences in the motor-related networks identified in this study may simply reflect the altered coherence characteristic of a resting state and may not directly predict dancer behavior. This is also an issue for all resting-state studies and requires further investigation. Multimodal and longitudinal designs may be useful in future investigations. In the current study, a cross-sectional design based on rest fMRI was adopted, which showed brain differences between the two groups. This design faced difficulties in its aim to reflect the brain plasticity resulting from dance training. Further longitudinal research should be included in the future.

## Conclusion

In conclusion, our findings reflect the enhanced functional connections in sensorimotor-related brain networks that exist in dancers who engage in prolonged modern dance training. The dancers presented with enhanced functional integration of cortico-basal loops and three biomarkers for optimal group separation with non-dancers, supporting the notion that improved motor execution and integration exists in dancers. In addition, the sensorimotor FC might predict the intensity of dance training. Moreover, the findings involving the putamen, which were closely related to rhythmical movement, indicate enhanced function related with dance properties. The findings derived from these tests offered substantial proof that the cortical ganglia loops of dancers operate at notably higher levels than for non-dancers. Although the results of this study were not outside the normal range compared to the existing research, they did contribute to our understanding of the benefits of dancing on the human brain.

Furthermore, dance is much more than a complex sensorimotor movement. It includes the experience and expression of emotions, the pleasure of esthetics and interpersonal communication. Our present research showed no evidence of esthetic or emotional intrinsic differences for dancers. These unconfirmed traits of dance may be a rich and promising field in future study.

## Additional Information

**How to cite this article**: Li, G. *et al*. Identifying enhanced cortico-basal ganglia loops associated with prolonged dance training. *Sci. Rep.* **5**, 10271; doi: 10.1038/srep10271 (2015).

## Supplementary Material

Supplementary Information

## Figures and Tables

**Table 1:** Information regarding the dancers.

**No.**	**Gender**	**Age**	**Started age**	**Training duration (years)**	**Average training time per week (hours)**
1	F	20	5	14	4
2	F	22	6	14	16
3	F	21	7	14	5
4	F	18	4	12	10
5	F	18	6	12	10
6	M	17	8	7	12
7	F	19	6	12	8
8	F	19	3	16	12
9	M	19	7	10	4
10	F	18	8	10	5
11	F	18	9	9	5
12	F	21	4	17	6
13	M	18	8	8	10
14	M	22	5	17	6
15	M	18	9	7	4
16	F	20	6	10	4
17	F	18	7	10	10
18	F	17	5	12	5
19	M	20	4	10	18
20	M	18	8	9	21
21	F	22	9	10	19
22	M	16	6	8	18
23	M	20	4	8	18
24	F	17	5	9	19
25	M	20	6	12	15
26	F	18	10	8	8
27	F	17	8	8	19
28	F	23	16	7	32

**Table 2:** The enhanced short- and long-range FCD regions in the group level (*p* < 0.001, cluster threshold *k* > 600 mm^3^).

	**Anatomical location**	**Cluster volume**	**MNI coordinate**			**Peak**
			[***X***	***Y***	***Z***]	***t***-score
Long-range FCD	Left Putamen	1728	[−28	9	4]	4.12
	Right Putamen	972	[30	10	−1]	3.75
	Right Superior Occipital Gyrus	918	[20	−88	21]	3.47
Short-range FCD	Left Postcentral Gyrus	1161	[−36	−25	53]	3.52
	Right Postcentral Gyrus	999	[36	-26	46]	3.43
	Right Precentral Gyrus	1323	[36	−20	53]	3.41
	Left Precentral Gyrus	1674	[−33	−15	54]	3.36

**Table 3:** The enhanced functional connections of seeds in the group level (*p* < 0.05 FDR-corrected, cluster threshold *k *> 600 mm^3^).

**Seed**	**Anatomical location**	**Cluster volume**	**MNI coordinate**			**Peak**
			[***X***	***Y***	***Z]***	***t***-score
Left Putamen	Right Middle Cingulate Gyrus	1107	[7	11	34]	4.75
Right Putamen	Right Middle Cingulate Gyrus	972	[9	8	35]	5.34
Left Postcentral	Right Postcentral Gyrus	5805	[24	−25	70]	4.98
	Right Precentral Gyrus		[23	−26	66]	4.68
	Right Middle Cingulate Gyrus	675	[4	−3	36]	4.76
	Left Postcentral Gyrus	2646	[−24	33	64]	4.56
	Left Precentral Gyrus		[−25	−24	68]	3.98
Right Precentral	Right Postcentral Gyrus	3186	[22	−36	69]	5.25
	Right Precentral Gyrus		[18	−26	67]	4.52
	Left Precentral Gyrus	1809	[−25	−23	69]	4.02
	Left Postcentral Gyrus		[−24	−34	67]	3.73
