# Supplementary material for: Identifying enhanced cortico-basal ganglia loops associated with prolonged dance training
Source: Sci Rep. 2015 Jun 2;5:10271. doi: 10.1038/srep10271 (PMC4649913; doi:10.1038/srep10271)
Supplement: Supplementary Information [file srep10271-s1.pdf]

Supplementary Materials For

Identifying enhanced cortico-basal ganglia loops associated with prolonged dance training

Gujing Li#, Hui He#, Mengting Huang, Xingxing Zhang, Jing Lu, Yongxiu Lai, Cheng Luo*, Dezhong Yao*

Key Laboratory for NeuroInformation of Ministry of Education, School of Life Science and Technology, University of Electronic Science and Technology of China

*Address correspondence to: Cheng Luo, No. 4, Section 2, North Jianshe Road, Chengdu, P.R. China, 610054. E-mail: chengluo@uestc.edu.cn, Tel: 86 28 61831272; Fax: 86 28 83206978

Dezhong Yao, No. 4, Section 2, North Jianshe Road, Chengdu, P.R. China, 610054. E-mail: dyao@uestc.edu.cn; Tel: 86 28 83201018; Fax: 86 28 83206978.

#These authors contributed equally to this work.

Region–wise functional connectivity in sensorimotor regions

To verify our findings that dance training induced functional plasticity in sensorimotor-related brain networks, we evaluated functional connectivity in sensorimotor regions. We selected seven regions of interest (ROI) including six regions (center coordinates were represented in Table 1) that revealed different FCD k-scores (the bilateral putamen, bilateral precentral gyrus and bilateral postcentral gyrus), and the middle cingulate cortex (MNI coordinate: $x = 7$, $y = 5$, $z = 35$) showed significant increased functional connections with seeds in the FC analysis (Fig. 2). Each ROI consisted of 27 voxels centered at the aforementioned coordinates. The mean time series of each ROI was extracted. Possible variances from the mean time series of ROIs were removed by regression, a general FC analysis procedure. The resulting time series were correlated between the ROIs of each subject. Then, the correlation coefficients were transformed to approximate a Gaussian distribution using Fisher's *z* transformation. The statistical analyses were performed on all possible connections represented in the correlation matrices between the dancer group and the non-dancer group.

To characterize the changes of FC between the dancer group and the non-dancer group; a further analysis was separately performed on a correlation coefficient (CC) of each edge, which was significantly FC between ROIs from the one-sample *t*-test statistical analysis in any group. Therefore, each single value for each participant was extracted. Then, a receiver operating characteristic (ROC) curve was generated to find an optimal threshold for classifying the two groups for each edge. Furthermore, each sensitivity, specificity and accuracy score was calculated.

Region–wise functional connectivity in sensorimotor regions in the **Dancers and the Non-dancers**

One-sample *t*-tests were performed on all possible connections represented in the correlation matrices within both groups. For better visualization of structural patterns within those connection matrices, a layout of ROIs and undirected edges were represented as networks (SFig. 1 a, b). The edges between

ROIs were constructed by setting the significance level of $p<0.01$. To compare the functional connectivity of each pair of ROIs between the two groups, two-sample two-tailed t-tests were performed on all potential connections included in the correlation matrices. Compared with the non-dancers, six pairs of correlations were significantly increased ($p<0.01$, FDR-corrected) in the dancer group (SFig. 1 c). No significant decrease was observed in the dancer group. Increased connection was primarily detected between the putamen and MCC. Increased connections were also detected between the MCC and right postcentral, as well as right precentral, gyri. There were enhanced functional connections between the precentral gyrus and the postcentral gyrus bilaterally.

In addition, in the ROC analysis of CC from significant edges, three cutoff values for the three edges could differentiate the dancer group and non-dancer group with sensitivity and specificity values exceeding 85% (SFig. 2, STable 1). In the functional connectivity between the right postcentral and right precentral, the cutoff value of 0.5 differentiated the dancers and non-dancers with 86% sensitivity and 88% specificity. In the FC between the right precentral and right middle cingulate cortices, the cutoff value of 0.11 differentiated the two groups with 86% sensitivity and 85% specificity. A similar result was found in the FC between the right postcentral and right middle cingulate cortices, the cutoff value of 0.13 differentiated two groups with 89% sensitivity and 85% specificity.

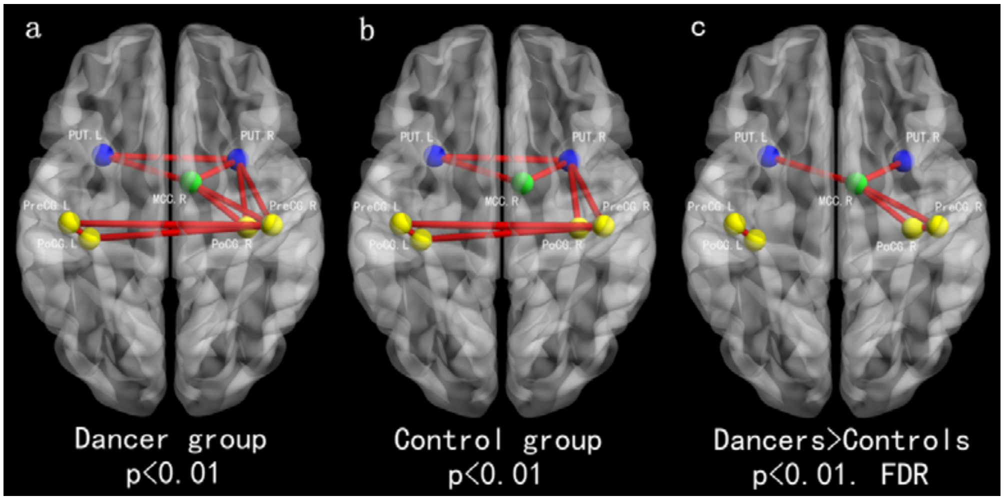

SFig. 1 Region–wise functional connectivity between sensorimotor regions

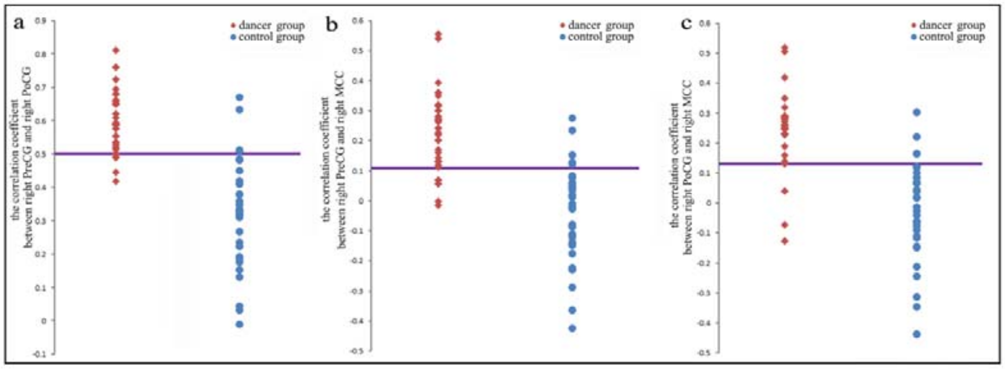

SFig. 2 Correlation coefficients from the significant FCs between ROIs. Part "a" represents the correlation coefficients between the right PreCG and the right PoCG. "b" represents the correlation coefficients between the right PreCG and the right MCC. "c' represents the correlation coefficients between the right PoCG and the right MCC.

STable 1. Classification performance

Functional Connectivity	Cutoff value	Sensitivity	Specificity	Accuracy
PreCG.R and PoCG.R	0.5	86%	88%	87%
PoCG.R and MCC.R	0.13	89%	85%	87%
PreCG.R and MCC.R	0.11	86%	85%	85%
PreCG.L and PoCG.L	0.49	71%	82%	77%
MCC.R and PUT.R	0.38	64%	73%	69%
PoCG.L and PoCG.R	0.52	61%	76%	68%
MCC.R and PUT.L	0.36	50%	85%	68%
PUT.R and PUT.L	0.46	82%	56%	62%
PreCG.R and PUT.R	0.28	14%	91%	57%
PoCG.L and PreCG.R	0.34	52%	75%	57%
PoCG.R and PUT.R	0.21	21%	85%	56%
PreCG.L and PoCG.R	0.36	60%	64%	56%
PreCG.L and PreCG.R	0.41	47%	61%	54%
